# The Rate of Decline of Glomerular Filtration Rate May Not Be Associated with Polymorphism of the PPAR**γ**2 Gene in Patients with Type 1 Diabetes and Nephropathy

**DOI:** 10.1155/2014/523584

**Published:** 2014-01-22

**Authors:** Bingmei Yang, Hongxin Zhao, Beverley Ann Millward, Andrew Glen Demaine

**Affiliations:** ^1^Molecular Medicine, Institute of Translational & Stratified Medicine, Plymouth University Schools of Medicine & Dentistry, John Bull Building, Tamar Science Park, Research Way, Plymouth PL6 8BU, UK; ^2^The Influenza Surveillance Section, Respiratory Diseases Department, Centre for Infectious Disease Surveillance and Control, Public Health England, London NW9 5EQ, UK

## Abstract

The aim of the study was to investigate whether a Pro12Ala polymorphism in the peroxisome proliferator-activated receptor gamma 2 (PPAR**γ**2) gene is associated with the progress of diabetic nephropathy in patients with type 1 diabetes. 197 Caucasian patients with type 1 diabetes and ethnically matched 151 normal healthy controls were genotyped for this polymorphism. Results showed that there were no significant differences in the frequencies of the genotypes and alleles of the polymorphism between groups. Multiple regression analysis in 77 patients demonstrated that the rate of decline in renal function in terms of glomerular filtration rate was significantly correlated to the baseline level of cholesterol (*P* = 0.0014), mean diastolic blood pressure during follow-up period (*P* = 0.019), and baseline level of HbA1c (*P* = 0.022) adjusting for the effect of diabetes duration and gender, but no significant association was found between the polymorphism and the progression of diabetic nephropathy in our studied population. In summary, our results show that the PPAR**γ**2 polymorphism is unlikely to be associated with the development and progression of the diabetic nephropathy in patients with type 1 diabetes. Further studies in different populations may be warranted to confirm our findings as the sample size in our study was relatively small.

## 1. Introduction

Peroxisome proliferation-activated receptor *γ* (PPAR*γ*) is a nuclear receptor which is an important regulator of adipocyte differentiation and a modulator of intracellular insulin-signalling events. There are two isoforms in PPAR*γ*: PPAR*γ*1 and PPAR*γ*2. PPAR*γ*2 is a ligand-activated transcription factor and a member of the steroid receptor superfamily. It is expressed in vascular smooth muscle cells, mesangial cells, and macrophages [1], which has been shown to be involved in lipid and glucose metabolism, fatty acid transport, and cell differentiation [2]. It is also highly expressed in renal epithelial cells [3, 4]. In addition, PPAR*γ* activation abrogated gene expression of metalloproteinase 9 (MMP-9) in murine macrophages [5, 6] and secretion of MMP-9 in the THP-1 human monocytic cell line [7]. These findings suggest that PPAR*γ* may play diverse roles in cell growth, differentiation, and extracellular matrix accumulation. The thiazolidinediones act as PPAR*γ* ligand [8, 9] and have been reported to decrease albuminuria in patients with early diabetic nephropathy [10]. This indicates a possible role of PPAR*γ* in the development of the diabetes-associated microvascular phenotype.

Ala12Prol substitution in exon B of the PPAR*γ*2 gene is within the domain of PPAR*γ*2 that enhances ligand-independent activation [11]. A functional study has shown that the Ala allele decreases the DNA-binding affinity of the PPAR*γ*2, thus reducing its transcriptional activity [12]. Several studies have demonstrated an association of this polymorphism with higher insulin sensitivity [12, 13], a decreased risk of type 2 diabetes mellitus [14] and its nephropathy [15–18] and retinopathy [19], and a lower albumin excretion rate in patients with established diabetes [15]. The Ala12 allele is also significantly associated with greater insulin sensitivity in childhood obesity [20] and in a large Italian population [21]. The mechanisms by which the PPAR*γ*2 Pro12Ala polymorphism could lead to diabetic nephropathy are unknown. A recent study has shown that the minor allele Ala12 of the PPAR*γ*2 gene may be a protective factor for type 1 diabetes mellitus [22]. However, it was observed that the Ala12 allele of the PPAR*γ*2 gene was associated with enhanced decline of renal function in patients with type 1 diabetes and nephropathy in Jorsal et al. study [23]. Those studies' results warrant further investigations and evidence; therefore, we conducted this study in attempt to further investigate the role of polymorphism of the PPAR*γ*2 gene in type 1 diabetes and nephropathy.

## 2. Materials and Methods

### 2.1. Subjects

A total of 210 patients (Caucasoid origin) with type 1 diabetes and with diabetic nephropathy (*n* = 120) or without microvascular complications (*n* = 90) were recruited into this study. 151 normal Caucasoid controls were also included into this study.

All patients with type 1 diabetes (as defined by The Expert Committee on the Diagnosis and Classification of Diabetes Mellitus [24]) had attended the Diabetes Clinic at Derriford Hospital, Plymouth, UK. Local Research Ethnics Committee approval was obtained. Informed consents were obtained from all subjects from whom blood was obtained prospectively. Normal controls were ethnically matched, Caucasoid cord blood samples following a normal, healthy obstetric delivery in the same hospital. The patients with type 1 diabetes were classified into different groups as previously described [25].

*Uncomplicated.* Patients have had type 1 diabetes for at least 20-years but remained free of retinopathy (fewer than five dots or blots per fundus) and proteinuria (urine Albustix negative on the consecutive occasions over 12 months). Nine patients with type 1 diabetes had not been diagnosed as having microvascular complications for less than 20-year duration of type 1 diabetes; therefore, they were excluded in further analysis. In total, there were 81 uncomplicated subjects included in the analysis.

*Nephropaths.* Patients have had type 1 diabetes for at least 10 years with persistent albuminuria (>300 mg/24 h) in two out of three consecutive measurements in sterile urines and retinopathy but no other kidney or urinary tract diseases [26]. We had four patients with persistent proteinuria for less than 10-year duration of type 1 diabetes; therefore, they were excluded in the analysis. In total, we had 116 patients with type 1 diabetes and diabetic nephropathy in this study.

Among those subjects with type 1 diabetes and diabetic nephropathy, complete retrospective clinical data was available in 77 patients. These clinical parameters have been retrieved from patients' hospital notes retrospectively since they were diagnosed with diabetic nephropathy. Estimated glomerular filtration rates (eGFR) were calculated with the Modification of Diet in Renal Disease equation [27]. The rate of decline in calculated GFR over time was calculated by linear regression analysis for the entire follow-up period for each of these patients. In addition to the analyses of the average or median changing trends of the calculated GFR, patients were also categorised into relatively fast and slow progression groups. In previous published studies, Andersen et al. [28, 29] demonstrated that their observed standard deviation (SD) on the rate of decrease in GFR in their studied group was 2.5 mL/min/year. They showed that an analysis with 54 subjects would detect a difference in the rate of decline in GFR of 2 mL/min/year (*α* = 5%, *β* = 20%) between two different ACE genotype groups. Therefore, with bigger sample size (77 subjects) in our study, we would have the power to detect a significant difference in the rate of the decline in GFR of 2 mL/min/year between the two different PPAR*γ*2 genotype groups.

### 2.2. Genotyping of PPAR*γ*2 Polymorphism

PCR was performed to amplify the unique PPAR*γ*2 exon from genomic DNA with the following amplifiers: forward 5′-CAAGCCCAGTCCTTTCTGTG-3′ and reverse 5′-CAGTGAAGGAATCG CTTTCCG-3′. The PCR products were digested with restriction endonuclease BstU 1 at 60°C for 2 hours and resulting products were electrophoresed through a 2.5% agarose gel, stained with ethidium bromide, and DNA visualized by ultraviolet transillumination. The expected products after digestion are 238 base pairs (bp) for Pro12Pro; 217 and 43 bp for Ala12Ala; and 238, 217, and 21 bp for Pro12Ala genotypes, respectively.

## 3. Statistical Analysis

The frequency of alleles and genotypes in the patients' subgroups and normal controls were compared using *χ* ^2^ test. Clinical data are reported as mean (±SD) or median (interquartile range). Comparisons between genotypes with respect to the continuous variables such as the changes of the calculated GFR, baseline cholesterol level, baseline haemoglobin A1c (HbA1c), and blood pressures were performed by use of Student's *t* test or the Mann-Whitney *U* test for the skewed data between comparing groups. *χ* ^2^ test was also used to compare the difference in proportions of genotypes between the relatively fast (i.e., the calculated GFR decline above or equal to the median of 2.48 mL/min/year) and slow (i.e., the calculated GFR decline below the median) progression groups. Multiple linear regression was used to examine the relationship between the PPAR*γ*2 genotype and various clinical and biochemical parameters for the decline of renal function. A *P* value of less than 0.05 (two-tailed) was considered to be significant.

The power of the test was calculated by using UCLA Binomial power calculation software. According to previous studies [30–33], current sample size would have at least 75% power at a 0.05 significance level to detect a difference between normal controls and patients with type 1 diabetes as well as between patients with nephropathy and patients without nephropathy.

## 4. Results and Discussions

There were no significant differences for age at onset of diabetes, duration of diabetes, and gender between groups (Table 1). The observed genotypes were consistent with Hardy-Weinberg equilibrium in type 1 diabetes and normal controls. There were no significant differences in frequencies of the alleles and genotype frequency between groups (Table 1). This was similar to previously published data [23, 34].

The details of clinical and genotype data obtained from the 77 patients with diabetic nephropathy are shown in Table 2. There were no significant differences detected between these Pro12Pro and Pro12Ala genotypes in their distribution patterns of gender, duration of diabetes, change in GFR, and other clinical indicators including HbA1c, cholesterol, and blood pressure either at baseline (the time of diagnosis of diabetic nephropathy) or during follow-up. There was no significant difference in the percentage of patients using ACE inhibitors and ARBs between the two genotype groups (data not shown).

The average change in GFR over time for the whole cohort of 77 patients was −3.9 mL/min/year and median (interquartile) was −2.48 mL/min/year (ranging from −6.7 to 0.9 mL/min/year). There was no significant difference in the change of GFR between the two genotypes Pro12Pro: −2.68 mL/min/year (median: interquartile) versus Pro12Ala: −2.20 mL/min/year (median: interquartile), *P* = 0.653 (Mann-Whitney *U* test) (Table 2). No significant differences were found in the distribution of genotypes between the slow and fast decline rate of GFR groups (cut-off value: −2.48 mL/min/year) (data not shown). As there is no standard way to categorise the decline rate of GFR into slow or accelerated GFR groups, we assigned patients either to the fast progression groups when patient's average annual decline of GFR was above or equal to the median value of 2.48 mL/min/year or to the slow progression group when patient's average annual decline of GFR was less than the median value of 2.48 mL/min/year. It is reasonable to use the median value of 2.48 mL/min/year as a cut-off value as this value is similar to the average value of the rate of decline of GFR, which was ~3 mL/min/year in patients with type 1 diabetes and proteinuria demonstrated in Andersen et al. studies [28, 29].

Simple regression analyses showed that the change in GFR was significantly reversely correlated to the baseline level of total serum cholesterol (*r* = −0.4, *P* = 0.0014), mean systolic blood pressure during follow-up (*r* = −0.36, *P* = 0.0037), mean diastolic blood pressure during follow-up (*r* = −0.41, *P* = 0.0008), and the mean arterial blood pressure during follow-up (*r* = −0.43, *P* = 0.0004) but not correlated to other clinical factors including patient age, diabetes duration, HbA1c at either baseline or during follow-up, total serum cholesterol level during the follow-up, and baseline blood pressure levels.

Multiple regression analysis of GFR with these parameters including PPAR*γ*2 Pro12Pro and Pro12Ala genotypes showed that the rate of decline in renal function (GFR) was significantly correlated to the baseline level of cholesterol (*P* = 0.0014), mean diastolic blood pressure during follow-up period (*P* = 0.019), and baseline level of HbA1c (*P* = 0.022) adjusting for the effect of diabetes duration and gender, but it was not correlated to PPAR*γ*2 Pro12Pro and Pro12Ala genotypes.

Studies on the Pro12Ala polymorphism of the PPAR*γ*2 gene have produced conflicting results in the development of diabetes and its complications from several groups in different ethnic populations [14, 33, 35–41] in patients with type 2 diabetes and nephropathy. Furthermore, two previous studies on type 1 diabetes produced disparate results [22, 42]. We did not find an association between Ala12 carriers and type 1 diabetes or diabetic nephropathy in the present study. Though several studies reported that the Ala12 allele protected patients from worsening albuminuria [15, 16] in type 2 diabetes and was associated with a higher GFR in metabolic syndrome population [43], they are contradicting with Jorsal et al. study [23] in patients with type 1 diabetes and diabetic nephropathy. Jorsal et al. observed that the Ala allele was associated with enhanced decline in GFR and predicts end-stage renal disease and all-cause mortality in patients with type 1 diabetes and nephropathy. The reasons for those discrepancies are not clear and might be due to ethnic differences and patients' heterogeneity between populations. In addition, sample size could be another contributor to those discrepancies between studies conducted by different research groups. We understand the limitation of our study due to a relatively small sample size. However, this research does add extra evidence to the literature on this topic for further research.

In conclusion, high blood pressure is a risk factor for the progression of diabetic nephropathy and no significant association was found between the PPAR*γ*2 Ala12Pro polymorphism and the progression of diabetic nephropathy in terms of GFR in our studied Caucasoid population.

## Figures and Tables

**Table 1:** Clinical characteristics and frequencies (%) of polymorphism of the PPAR*γ*2 gene in patients with type 1 diabetes and normal controls.

	Uncomplicated (*n* = 81)	Nephropaths (*n* = 116)	All patients (*n* = 197)	Normal controls (*n* = 151)
Male : Female	31 : 50	52 : 64	84 : 114	64 : 87
Age at onset of diabetes	17.1 + 11.0 (2–51)	15.2 + 9.3 (1–54)		
Duration of diabetes	32.2 + 9.9 (20–65)	30.4 + 9.9 (13–65)		
Genotype				
Pro12Pro	65 (80.2)	90 (77.6)	155 (78.7)	125 (82.8)
Pro12Ala	14 (17.3)	25 (21.6)	39 (19.8)	23 (15.2)
Ala12Ala	2 (2.5)	1 (<1)	3 (1.5)	3 (2.0)
Allele				
Pro12	144 (88.9)	205 (88.4)	349 (88.6)	273 (90.4)
Ala12	18 (11.1)	27 (11.6)	45 (11.4)	29 (9.6)

**Table 2:** Genetic data, baseline and follow-up clinical data (means ± SD), and results of comparing these indicators between the PPAR*γ* genotypes in 77 patients with type 1 diabetes and nephropathy.

Parameters	All patients	PPAR*γ*2 genotype		*P* value
		Pro12Pro	Pro12Ala	
Number (male/female)	77 (34/43)	62 (26/36)	15 (8/7)	0.425
Age (years)	42 ± 13	43 ± 14	38 ± 9	0.206
Diabetes duration (years)	27 ± 9	27 ± 10	25 ± 8	0.519
Change in GFR (mL/min/year)	−3.9 ± 9.3 (−6.7 to 0.9)	−3.6 ± 9.7 (−6.6 to 1.0)	−4.9 ± 8.2 (−7.16 to 0.69)	0.629
Baseline data				
HbA1c (mmol/mol)	88.0 ± 6.0	85.8 ± 4.9	95.6 ± 9.3	0.273
Cholesterol (mmol/L)	6.0 ± 1.4	5.9 ± 1.3	6.6 ± 1.5	0.101
Systolic BP (mmHg)	143 ± 25	141 ± 22	151 ± 32	0.181
Diastolic BP (mmHg)	82 ± 13	81 ± 12	84 ± 16	0.465
Mean arterial BP (mmHg)	102 ± 15	101 ± 14	106 ± 19	0.261
Follow-up data				
HbA1c (mmol/mol)	78.1 ± 1.6	80.3 ± 0.5	71.6 ± 8.2	0.313
Cholesterol (mmol/L)	5.6 ± 1.5	5.8 ± 1.1	4.9 ± 2.3	0.179
Systolic BP (mmHg)	140 ± 19	140 ± 18	139 ± 21	0.952
Diastolic BP (mmHg)	78 ± 9	79 ± 9	78 ± 10	0.776
Mean arterial BP (mmHg)	99 ± 11	99 ± 11	98 ± 13	0.849

BP: Blood pressure; GFR: glomerular filtration rate; HbA1c: haemoglobin A1c.
